# Structural Diversity in Conserved Regions Like the DRY-Motif among Viral 7TM Receptors—A Consequence of Evolutionary Pressure?

**DOI:** 10.1155/2012/231813

**Published:** 2012-07-30

**Authors:** Ann-Sofie Mølleskov Jensen, Alexander Hovard Sparre-Ulrich, Nicholas Davis-Poynter, Mette Marie Rosenkilde

**Affiliations:** ^1^Laboratory for Molecular Pharmacology, Department of Neuroscience and Pharmacology, The Panum Institute, University of Copenhagen, Building 18.5, Blegdamsvej 3, 2200-Copenhagen N, Denmark; ^2^Sir Albert Sakzewski Virus Research Centre (SASVRC), Royal Children's Hospital/Clinical Medical Virology Centre (CMVC), University of Queensland, St Lucia, QLD 4072, Australia

## Abstract

Several herpes- and poxviruses have captured chemokine receptors from their hosts and modified these to their own benefit. The human and viral chemokine receptors belong to class A 7 transmembrane (TM) receptors which are characterized by several structural motifs like the DRY-motif in TM3 and the C-terminal tail. In the DRY-motif, the arginine residue serves important purposes by being directly involved in G protein coupling. Interestingly, among the viral receptors there is a greater diversity in the DRY-motif compared to their endogenous receptor homologous. The C-terminal receptor tail constitutes another regulatory region that through a number of phosphorylation sites is involved in signaling, desensitization, and internalization. Also this region is more variable among virus-encoded 7TM receptors compared to human class A receptors. In this review we will focus on these two structural motifs and discuss their role in viral 7TM receptor signaling compared to their endogenous counterparts.

## 1. Introduction

Seven transmembrane (7TM) receptors constitute the largest superfamily of membrane proteins and function as important mediators of extracellular signals to intracellular responses. The chemical diversity of the endogenous ligands is tremendous ranging from small simple chemical entities like photons, ions, and nucleotides, to more complex small ligands like monoamines and peptides, and larger proteins, glycoproteins, and lipids. The 7TM receptors are divided into five classes of which class A or rhodopsin-like receptors is the dominating class [1]. The receptors are characterized by seven membrane-spanning *α*-helices as well as coupling to G proteins; hence, the name is G protein coupled receptors (GPCRs). (In this review we will use the term 7TM receptors instead of GPCRs as these receptors also signal trough non-G protein-dependent pathways, like **β**-arrestin-mediated signaling [2].) Signaling by 7TM receptors through G proteins leads to, for example, either inhibition (G_*αi*_) or activation (G_*αs*_) of adenylyl cyclase and cAMP production, activation of phospholipase C with inositol triphosphate turnover (G_*αq*_), or activation of RhoGEF (G_*α*12/13_) depending on which G protein the receptor is activating [3]. Furthermore, the G_*βγ*_ subunit is also involved in signaling and the 7TM receptors also signal via G protein-independent pathways like MAP-kinase activation-mediated by *β*-arrestins [4].

Despite the structural diversity in the repertoire of the endogenous 7TM receptor agonists, the conformational changes that occur upon receptor activation are believed to be overall identical. Thus, as the last two decades of biochemical and biophysical studies indicate, TM6, and to a minor degree TM7 and TM3, undergo conformational rearrangement during receptor activation [5, 6]. Centered around the highly conserved proline in the middle of TM6 (position VI:15 or 6.50) TM6 is believed to perform movements that results in space creation thereby permitting binding of intracellular signal transduction molecules like G proteins and *β*-arrestins [7]. (The numbering of amino acids in the helices is provided according to two numbering systems: the generic numbering system suggested by Schwartz [8], followed by the numbering system of Ballesteros and Weinstein [9],) Several crystal structures of 7TM receptors have been presented within the last decade initiated by the structure of bovine rhodopsin [10] followed by the adrenergic receptors [11–15], the adenosine receptors [16–18], additional rhodopsin variants [19–21], muscarinic receptors, [22, 23] and several others [24–26] including the chemokine receptor CXCR4 [27]. In the recent years, crystal structures of not only inactive, but also active 7TM receptors, have been identified. Thus, in the agonist-bound *β*2-adrenergic receptor, a relatively large rearrangement of the lower segments of TM6 is observed, when compared to the corresponding inactive structure [13–15]. This structural feature is also observed in the crystal structure of opsin in complex with a G protein peptide fragment upon comparison with dark-state rhodopsin [21, 28]. The overall arrangement of the seven transmembrane *α*-helices delineate the main binding pocket, and most studies in the search of functionally important residues have focused on amino acids facing this main binding pocket (delimited by TM3, TM4, TM5, TM6, and in part TM7). This is with good reason as most small molecule ligands interact with residues in this pocket [11, 12, 29]. Furthermore, most conserved microswitches of functional importance also face the main binding pocket. This includes ArgIII:26 (3.50), which is part of the conserved DRY-motif in TM3, the rotameric toggle switch TrpVI:13 (6.48), which is part of the CWxP-motif in TMVI, and TyrVII:20 (7.53), which is part the NPxxY-motif in TMVII—all of which play crucial roles during receptor activation [30, 31]. However, also residues in the region delimited by TM1, TM2, TM3, and TM7 (the so-called minor binding pocket) function as regulatory switches or major ligand anchor points [32–34]. 

The DRY-motif is the most conserved motif among the microswitches mentioned above (Figures 2(a) and 2(c)) [21, 30] and has been shown to directly interact with the G protein in a recent crystal structure of the *β*2-adrenergic receptor in complex with the G_*αs*_-subunit—a crystal that displayed the actual signaling complex and uncovered the importance of both the DRY-motif and the NPxxY-motif in receptor activation [14, 35]. While the overall interaction between the G protein and the receptor is mainly hydrophobic within the transmembrane core, the ArgIII:26 (3.50) is sandwiched between a Tyr in the G protein and TyrVI:20 (7.53) of the NPxxY-motif, highlighting the importance of concerted action of both motifs [14, 35].

The positively charged ArgIII:26 (3.50) has been proposed to be involved in other conformational constrains of importance for receptor activation. Thus, an inactivating salt bridge (a so-called ionic lock) has been suggested between the Arg and another conserved residue, the acidic GluVI:-05 (6.30) in intracellular loop 3 (ICL3) [36]. This ionic lock is broken during receptor activation where TyrVI:20 (7.53) rotates towards the helix bundle as seen in the active crystal structures of both rhodopsin [21] and the *β*2-adrenergic receptor irreversibly bound to an agonist [37] or stabilized by a nanobody [13]. However, as the GluVI:-05 (6.30) is only conserved among 25 % of all class A receptors [14, 35], and not present in any of the chemokine receptors [38], the molecular interactions involved in conformational constraining of inactive receptor states and the role of ArgIII:26 (3.50) must be different in receptors without GluVI:-05(6.30). Finally, the DRY-motif interacts with ICL2 of the receptor, thereby stabilizing a position of this loop capable of interacting with a hydrophobic pocket on the G protein and directly linking the highly conserved DRY-motif to the receptor/G protein interaction [14].

Anotherimportant region for receptor activity is the intracellular C-terminal tail of the 7TM receptors as it contains phosphorylation sites and other regulatory recognition motifs necessary for desensitization by G protein-coupled receptor kinases (GRKs), *β*-arrestin recruitment and signaling, internalization and receptor recycling, and for other means of signal regulation [39]. These two receptor motifs will be the focus of the current review, where we will compare the structural and functional properties, degree of conservation, and functional diversity of the two motifs between class A 7TM receptors encoded by viruses and endogenously encoded 7TM receptors. Most of the virus-encoded 7TM receptors belong to the chemokine subfamily [40] and consequently extra attention will be directed towards the viral molecular piracy within the chemokine system and the endogenous chemokine receptors.

## 2. The Chemokine System

The chemokine system plays an important role in the human immune defense against pathogens such as viruses since the chemokines (abbreviated from chemotactic cytokines) are involved in leukocyte migration during inflammation and also control activation and differentiation of lymphoid cells [41, 42]. The chemokine receptors belong to class A 7TM receptors and comprise the largest subfamily within this group with 19 different endogenous chemokine receptors and up to 50 chemokine ligands [43]. The chemokines are divided into four subfamilies depending on the presence or absence of residues between the first two of usually four conserved cysteines: the CXC (CXCL1-16) and CC (CCL1-28) chemokines along with the CX3C (CX3CL1) and XC (XCL1) chemokines [44, 45]. The CXC chemokines are further divided based on an ELR-motif prior to the CXC-motif. (In the following, the “novel” systematic chemokine nomenclature is used [44]) ELR CXC chemokines are induced under acute and chronic inflammation, play an angiogenic role, and mainly attract neutrophils while the non-ELR CXC chemokines exert their effect on lymphocytes and are more constitutively expressed as well as being angiostatic or angiomodulatory [46, 47]. The chemokine receptors are likewise divided into four groups in accordance with the classification of their preferred ligands [44, 48]. The interaction between chemokine and receptor range from high selectivity to large promiscuity; although not cross-interacting with other subfamilies. The main signaling pathway of the endogenous chemokine receptors is via G_*αi*_ leading to calcium release and chemotaxis [49].

## 3. The Virus-Encoded 7TM Receptors

Considering the role of chemokines in the immune system it is not surprising that several viruses, by an act of molecular piracy of host genes, encode chemokines and/or chemokine receptors in their genomes. It is primarily the large poxviruses and the *β*- and *γ*-herpesviruses which encode chemokine receptors (and ligands) [50], however also the retrovirus HIV utilizes the endogenous chemokine system by using the two chemokine receptors CCR5 and CXCR4 as cell-entry co-factors together with CD4 during infection and spread [42, 51]. Also the viral CC (and CX3C) chemokine receptor homolog US28 (described further below) encoded by HCMV (human cytomegalovirus) has been implicated as a HIV cell-entry co-factor [52]. The majority of the viral receptors have structural features in common with the endogenous chemokine receptors in spite of having a sequence identity to these of only 25–59% [42]. However, compared to the endogenous chemokine receptors, the viral receptors show a vast divergence in their signaling capacities as well as ligand specificity with constitutive activity being typical for the viral receptors, unlike the endogenous chemokine receptors [53–57]. Constitutive activity also occurs among endogenous non-chemokine receptors, as shown for a few receptors [58]; however an increasing number of examples illustrate that the range of naturally-occurring constitutively activating mutations are tightly associated with disease or a particular phenotype [58]. This includes mutations in the melanocortin 1 receptor (MC1R; associated with melanism, [59]), MC4R (obesity, [60]), the ghrelin receptor (short stature, [61]) and rhodopsin (retinitis pigmentosa, [62]) among others. 

Furthermore, besides being constitutively active, the viral receptors also signal promiscuously through many pathways, as compared to the predominant G_*αi*_ coupling of endogenous chemokine receptors [41, 47]. For instance the ORF74 (open reading frame 74) 7TM receptor encoded by HHV8 (human herpesvirus 8) associates with both G_*αi*_ and G_*αq*_ [63] as well as signals through MAP kinases [64] leading to the activation of numerous transcription factors, cell proliferation and transformation, VEGF secretion and angiogenesis [64–68]. The ORF74 from herpesvirus saimiri (HVS-ECRF3) also signals through both G_*αi*_, G_*α*12/13_, and G_*αq*_ in a ligand-dependent manner, however the constitutive activity of this receptors is constrained to G_*αi*_ and G_*α*12/13_, but not G_*αq*_ [69, 70]. A similar broad spectrum and promiscuous signaling is also observed for the US28 (unique short 28) and UL33 (unique long 33) 7TM receptors encoded by HCMV, which signals constitutively through both G_*αi*_, and G_*αq*_ along with MAP kinases [71–73].

**Figure 1:**
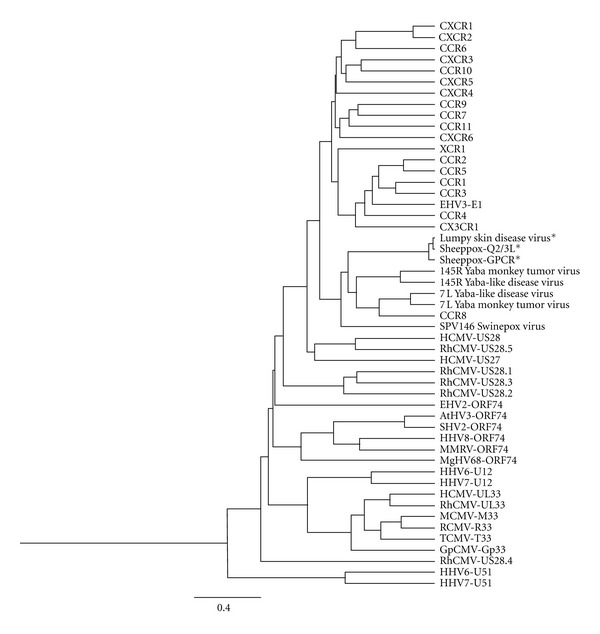
Phylogenetic tree of viral and human chemokine receptors based on their amino acid sequence. The length of each branch reflects the similarity between receptors. It was generated by aligning the sequences using the settings: Blosum62, gap open penalty of 5 and gap extension penalty of 0.1 followed by the Jukes-Cantor distance analysis done in Geneious Pro. For further information about the virus-encoded receptors and GenBank accession number please see Table 1. *As the sequence of these viral receptors are very alike it cannot be excluded that they are in fact the same.

Besides being evolutionary distinct from the endogenous chemokine receptors, the herpesvirus-encoded chemokine receptors cluster in four families (Figure 1): U12/UL33 of HHV6, HHV7, and CMV; U51/UL78 of HHV6, HHV7 and CMV; US27/US28 of CMV; and ORF74 of HHV8 as well as non-human herpesviruses [50]. (UL78 from CMV is evolutionarily conserved with U51 from HHV6 and HHV7. However, as the UL78 receptors have shown no functional homology to chemokine receptor, they have been excluded from current review.) The common feature of encoding chemokine receptors throughout the pox- and herpesviruses suggests that these receptors play an important role in the viral life cycle as well as in circumvention of the host immune system. A few studies of receptor-disrupted viruses have shown diminished replication *in vivo* in selected tissues [74, 75].

Chemokine receptors are also found in the genomes of poxviruses [76, 77]. In contrast to the broader subfamily resemblance to CC as well as CXC chemokine receptors along with the promiscuous chemokine-binding profile of many herpesvirus-encoded receptors, the poxvirus-encoded receptors solely resemble the CCR8 chemokine receptor (as illustrated in Figure 1) and only interact with CCR8-binding ligands [78, 79]. Briefly, the poxvirus-encoded receptors are located in two areas in the viral genome: 7L and 145R. The best characterized poxvirus receptors are 7L and 145R from YLDV (Yaba-like disease virus) [76, 77].

Also, nonchemokine receptors are found in viral genomes exemplified by the BILF family from several *γ*1-herpesviruses including human EBV (Epstein Barr virus). The BILF1 receptors from human and rhesus EBV are the only BILF receptors that have been characterized from a pharmacological point of view and like most other virus-encoded 7TM receptors they display constitutive activity [80, 81]. As an extra refinement and interplay with the host immune system, it should be mentioned that viruses also regulate the expression of endogenous 7TM receptors within the chemokine system, and class A in general. For instance, GPR183, also known as EBI2 (Epstein-Barr virus induced receptor 2), which is induced >200 fold upon EBV cell-entry [33, 40, 82].

## 4. The Impact of the DRY-Motif among ****Endogenous Class A Receptors

**Figure 2:**
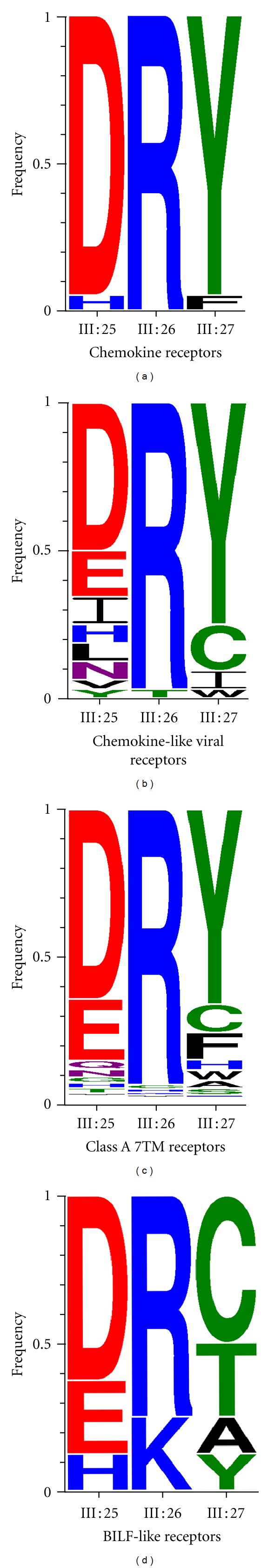
Sequence logos of the DRY-motif for chemokine receptors (a), chemokine-like viral receptors (b), class A 7TM receptors (c), and BILF-like receptors (d). The chemical properties of the amino acids are represented in color (polar: green, neutral: purple, basic: blue, acidic: red, and hydrophobic: black). This figure was created using the web application: http://weblogo.threeplusone.com/.

The Asp, Arg, Tyr, or DRY-motif in the intracellular end of TM3 is one of the most conserved motifs among class A 7TM receptors [31] (Figure 2(c)) and plays a pivotal role in receptor activation. This amino acid triplet is located in positions III:25 (3.49), III:26 (3.50), and III:27 (3.51), respectively, at the border to the intracellular loop 2 (ICL2) with a conservation as DRY in 66%, 96%, and 67% of all class A 7TM receptors [31]. The DRY-motif is even more conserved within the chemokine subfamily (Figure 2(a)) with 100% conservation of ArgIII:26 (3.50) and 95% conservation of both aromatic residues in position III:27 (3.51) and negatively charged residues in position III:25 (3.49). 

As mentioned in the introduction, it has been suggested that, in some class A 7TM receptors (e.g., the *β*-adrenergic receptors and in rhodopsin) the ArgIII:26 (3.50) together with AspIII:25 (3.49) and GluVI:-05 (6.30), the latter located in ICL3, form an ionic lock holding TM3 and TM6 together in the inactive state [10, 36]. During receptor activation, protonation of AspIII:25 (3.49) leads to release of the constraining interaction, thus allowing the outward movement of TM6 [5, 83]. This is also supported by charged- neutralizing mutations of AspIII:25 (3.49) suggesting that this residue is important for receptor activation [84]. However, the negatively charged residue at position VI:-05 (6.30) is not nearly as conserved as the DRY-motif indicating other possible ways to constrain the receptor in the inactive conformation [30]. During receptor activation, the interaction between adjacent Asp/GluIII:25 (3.49) and ArgIII:26 (3.50) of the DRY-motif is disturbed and ArgIII:26 (3.50) is instead able to interact with TyrV:24 (5.58) as well as directly with the G_*α*_ protein. This direct interaction with the G protein has been confirmed by the crystal structure of Opsin in complex with a small peptide from the C-terminal of the G_*αt*_ protein [21]. Receptor activation opens a pocket at the intracellular site making the interaction with the C-terminal of the G_*α*_ protein possible. This allows for the exchange of GDP with GTP thus activating the G protein for further downstream signaling [85].

From the crystal structure of the chemokine receptor CXCR4, it is evident that the overall structure is similar to the other crystal structures of class A 7TM receptors, nevertheless with a few differences mainly in the extracellular part which constitutes the chemokine ligand-recognition domain [27]. Importantly, the chemokine receptors (though belonging to class A) contain a *positively charged* residue at position VI:-05 (6.30) and hence the ionic lock between ArgIII:26 (3.50) and a negative charged residue at VI:-05 (6.30) does not exist in these receptors [38]. The lack of a negative charge in this position has inspired the introduction of the ionic lock in the chemokine receptors by substitution of the positive charge in VI:-05 (6.30) with a negative charge. This introduction of the putatively correct conditions for the ionic lock resulted in a reduced basal activity of the chemokine receptor CCR5, indicating that the receptor is locked in an inactive conformation with no ligand-induced activation and a strongly impaired ability to bind chemokines. Substitution of ArgIII:26 (3.50) with Ala or Gln maintained chemokine binding though still showed reduced basal activity [38]. Thus, the presence of an ionic lock between TM3 and TM6 leaves the chemokine receptor unable to switch to an active conformation; therefore, chemokine receptors must utilize a different mechanism than the classic ionic lock described above. However, the decrease in basal activity of CCR5 upon introduction of the ionic lock is in accordance with the general interpretation of the role of this motif in class A receptor activation, where loss of the ionic lock (VI:-05 (6.30) mutation), leads to constitutive activity [36]. Disregarding the lack of an ionic lock in chemokine receptors, the DRY-motif still plays an important role in the receptor activation, as exemplified in CCR5, where mutation of ArgIII:26 (3.50) to the neutral Asn disrupted both basal activity and chemokine-induced G_*αi*_ protein coupling (through calcium mobilization and GTP*γ*S binding assays) despite retained affinity for CCL4 [86]. Interestingly, an increased basal phosphorylation of the ArgIII:26Asn- (3.50-) mutated receptor was observed along with *β*-arrestin-mediated endocytosis as well as a higher rate of internalization in response to CCL4 stimulation [86]. Other studies have indicated the need for an intact DRY-motif in *β*-arrestin1 binding to CCR5, thus supporting the importance of this motif for receptor function [87].

Another class A receptor lacking the ionic lock residue VI:-05 (6.30) is the histamine 4 receptor (H4R), which shows high constitutive activity. Introduction of GluVI:-05 (6.30) did, however, not decrease the constitutive activity [88] as expected from other studies where disruption of an already existing ionic lock leads to increased constitutive activity [36] or like the CCR5 chemokine receptor loss of activity upon ionic lock introduction [38]. Additionally, the H3R also shows constitutive activity in spite of having the putative conditions for an ionic lock [88–90] indicating that the histamine receptors have a functional difference from the general class A 7TM receptors when it comes to activation and constitutive activity. However, the H4R did show complete loss of G protein activation upon mutation of ArgIII:26 (3.50) supporting the importance of the DRY-motif for coupling to G proteins [85, 88, 91]. A similar phenomenon was observed in the H2R where charge-neutralizing mutations of ArgIII:26 (3.50) led to severely decreased basal cAMP production in terms of efficacy indicating diminished G_*αs*_ coupling. However, the mutated receptor was able to induce a response upon agonist stimulation though still with lower efficacy compared to wild type [92]. In the same study, it was shown that the charge-neutralizing mutations of ArgIII:26 also resulted in highly structurally instable receptors, where surface expression could only be detected after stabilization with either an agonist or inverse agonist, indicating a role not only in receptor activation, but also in receptor stability for this position [92]. Furthermore, the DRY-motif has also been implicated in receptor stability of the *β*2-adrenergic receptor [15].

The C5A binding protein, C5L2, is among the few 7TM receptors that lack a positive charge in position III:26, as it has a Leu in this position (together with Asp in III:25 (3.49) and Cys in III:27 (3.51) [93]). The native C5L2 is also known as a nonsignaling C5A binding protein, however, this impaired G protein coupling could be partially restored by reintroducing ArgIII:26 (3.50) [93]. Other endogenous class A receptors without a positive charge in III:26 (3.50) include D6 and Duffy antigen/receptor for chemokines (DARC)—two nonsignaling 7TM structured receptors belonging to the chemokine receptor system. Both are known as nonsignaling proteins as they do not couple to G proteins, but they exert chemokine scavenging and transendothelial transport instead. DARC is, furthermore, rather specifically expressed by endothelial cells lining postcapillary venules, and here it exerts its presumed role in accumulation of extravascular chemokines, chemokine transcytosis, and presentation on the luminal surface thereby facilitating leukocyte adhesion [94–97]. GPR77, GPR78, and GPR133 constitute three orphan class A receptors with unknown functions that also lack the ArgIII:26 (3.50), and, in addition a handful of receptors without positive, charge are identified among the olfactory receptors.

## 5. The DRY-Motif Is Less Conserved among ****Virus-Encoded 7TM Receptors

The conservation of the ArgIII:26 (3.50) in the DRY-motif is low among the virally encoded chemokine receptors when compared to the endogenous counterparts (Figures 2(a) and 2(b)). Furthermore, there is a much larger diversity with respect to all three residues as evident by Figure 2(b). These changes in the DRY-motif could be part of the reason for the altered signaling properties with higher constitutive activity, and activation of a broader range of signaling pathways [53, 98]. 

Regarding ArgIII:26 (3.50), one receptor deserves special notice, namely, the CXC chemokine receptor ORF74 from equine herpesvirus 2 (EHV2). This receptor contains a DTW-motif instead of the DRY consensus, thus missing a positive charged residue in position III:26 (3.50). In spite of this, the receptor shows constitutive activity through the G_*αi*_ pathway and ligand-mediated signaling in response to the endogenous chemokine CXCL6 [55, 99]. Interestingly, introduction of the DRY-motif in the EHV2-ORF74 led to a 4-fold decrease in constitutive activity while retaining activation by the agonist, CXCL6 [55], suggesting that this receptor has been optimized to act in the absence of a positive charge in the DRY-motif.

As is also evident from Figure 2(b), the virus-encoded chemokine receptors from different pox- and herpesviruses show a larger diversity in the whole DRY-motif; primarily with large deviations within the first residue III:25 (3.49). For instance, the CXC chemokine receptor ORF74 by HHV8 contains a VRY-motif in place of the endogenous DRY-motif. This receptor is associated with Kaposi's sarcoma and shows a high degree of constitutive signaling and stimulates proliferation [100] as well as tumor transformation in mice [64]. The closest endogenous chemokine receptor to HHV8-ORF74 is CXCR2, and studies have shown that replacement of AspIII:25 (3.49) with Val in CXCR2, thus making this receptor more ORF74-like with respect to this motif, leads to constitutive activation of CXCR2 with altered signaling properties in the direction of HHV8-ORF74 signaling [101]. In contrast, the opposite mutation in HHV8-ORF74 (ValIII:25Asp thereby reintroducing the DRY-motif) did not have major effects on either ligand binding or receptor signaling [102]. Another example is the ORF74 receptor from murine herpesvirus 68 (MHV68), which contains an HRC-motif and has been indicated to activate similar oncogenic pathways as HHV8-ORF74 [103, 104]. However, further studies are needed to determine the signaling capabilities of several ORF74 receptors as well as the influence of their altered DRY-motif on constitutive activity and regulatory circumvention. 

The human herpesviruses HHV6 and HHV7 both encode two 7TM receptors, U12 and U51, which contain IRY- and ERI-motifs, respectively. HHV6-U12 has been shown to act as a chemokine receptor [105], whereas HHV6-U51 has been shown to be a constitutively active G_*αq*_-coupled CC chemokine receptor [106]. However, neither of these two receptors have the functional impact of the altered DRY-motif been studied. 

The UL33 family, consisting of 7TM receptors from murine (M33), rat (R33), and human (UL33) cytomegalovirus, is known to constitutively signal through a vast array of G proteins [72]. The rodent counterparts differ from the human by containing an NRY-motif whereas UL33 contains the conserved DRY-motif. A mutational analysis of M33 revealed that ArgIII:26 (3.50) is important for the viral constitutive signaling in NFAT, CREB, and IP-turnover assays as mutation into a neutral Gln abolished constitutive activation of the receptor. The importance of this was supported by *in vivo* data where a virus with a missing ArgIII:26 (3.50), NRY changed to NQY, was unable to replicate in the salivary glands [107]. Very interestingly, mutation of AsnIII:25 (3.49) into the consensus AspIII:25 (3.49), NRY to DRY, leads to an increased constitutive signaling (especially through NFAT-mediated transcription) suggesting that the endogenous DRY-motif is preferable for high activity. Having *lower* receptor activity could be advantageous for the virus as this might be favorable for the virus life cycle [107]. In line with the results for M33, a mutation of ArgIII:26 (3.50) rendered the R33 receptor inactive with respect to G protein coupling [108]. However, in contrast to M33, it was found that replacement of AsnIII:25 (3.49) with the endogenous AspIII:25 (3.49) in R33 (NRY to DRY) did not change the constitutive activity, and that replacement with the nonpolar AlaIII:25 (3.49) (NRY to ARY) led to a diminished PLC stimulation, but an unaltered pertussis toxin-sensitive signaling indicating impaired G_*αq*_-signaling but maintained G_*αi*_. 

Another HCMV-encoded receptor, US28, signals constitutively through several pathways such as G_*αq*_/phospholipase C, NF*κ*B, CREB, and MAP kinases [73, 109, 110]. This viral chemokine receptor contains the conserved DRY-motif and a mutational analysis of ArgIII:26Ala found that disruption of the DRY-motif leads to impaired G_*αq*_ protein activation and IP-turnover in spite of wild-type levels of cell-surface expression [111]. Like HHV8-ORF74, US28 has been implicated in cancer as the constitutive signaling of the receptor can activate proliferative pathways leading to tumor formation [112–114]. Additionally, HCMV has been found in glioblastomas, which could indicate a possible role in tumorigenesis [115–118].

Also among the BILF receptors, found in several *γ*1-herpesviruses, has this motif obtained extra attention. Interestingly, the DRY-motif in the constitutively active BILF1 receptors from human and rhesus EBV differs from the consensus in all three residues being EKT (GluIII:25 (3.49), LysIII:26 (3.50), ThrIII:27 (3.51), Figure 2(d)) [80, 81]. Substitution of EKT with EAT in BILF1 from EBV resulted in abolished G_*αi*_ signaling, whereas the conservative substitution of Lys with Arg (ERT) signaled as wt BILF1 (EKT). Interestingly, introduction of the whole conserved motif (DRY) actually impaired the receptor activity partially, indicating that the EKT-motif is functionally superior to the conserved DRY-motif in this BILF1 receptor. In addition to the G_*αi*_ coupling, the authors tested the impact of the EAT-motif in NIH3T3 cell transformation and tumor growth in nude mice, and they found that also via these pathways the EAT-motif was completely silent compared to wt BILF1 (EKT). Furthermore, the DRY substitution displayed an intermediate active phenotype in these two functional readouts [119]. Thus, the BILF1 receptor depends on a positive charge in the DRY-motif and has, as a consequence of the altered motif (DRY to EKT), been optimized to signal with higher activity [80, 81, 119].

## 6. The C-Terminal Tails of Class A Receptors ****Are Conserved with Respect to Length and ****Number of Phosphorylation Sites

**Figure 3:**
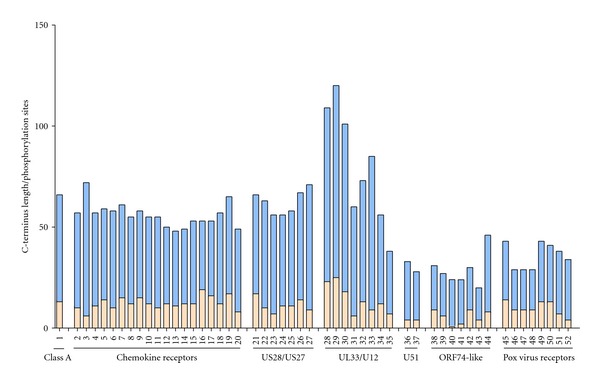
Relative sequence length and putative phosphorylation sites of the C-terminal region of class A 7TM receptors, chemokine receptors and virus-encoded chemokine receptors. The vertical axis displays the number of amino acids of the C-terminus (blue), defined as being after the highly conserved proline of the NPxxY-motif, and the number of serine, threonine and tyrosine in this region (beige). The horizontal axis displays the average of 334 non-olfactory class A 7TM receptors (1), the chemokine receptors (2–20) and the viral chemokine receptors (21–52). For further information about specific receptors and GenBank accession number please see Table 1.

Whereas the extracellular N-terminal region of class A 7TM receptors are quite diverse, the intracellular C-terminal tails are more homologous, both in terms of length and primary structure. As evident from Figure 3, the average length and number of phosphorylation sites are similar among the endogenous chemokine receptors and class A 7TM receptors in general, whereas the virally encoded receptors show a larger diversity, but are generally shorter in length and have fewer phosphorylation sites. The phosphorylation sites serve important regulatory purposes for receptor desensitization and cell surface expression [120, 121]. Quickly after receptor activation and G protein interaction, GRKs initiate phosphorylation of serine and threonine residues in the C-terminal tail (and intracellular loops) thereby promoting the interaction of the receptor with *β*-arrestins and a subsequent steric hindering of the receptor/G protein-interaction [4]. The consequences of *β*-arrestin recruitment are endocytosis of the receptor/*β*-arrestin complex and a subsequent recycling to the cell surface or degradation. Interestingly, *β*-arrestins only associate with the ubiquitin ligase promoting the degradation pathway when it interacts with a ligand-stimulated receptor [122, 123]. The shorter C-terminal tails of the viral receptors could suggest that viruses circumvent the host regulatory processes of receptor internalization in order to obtain constitutive signaling abilities. Furthermore, some viral receptors are constitutively endocytosed and predominantly intracellularly localized [74, 124, 125], which have led to the suggestion, that they could act as scavengers (like DARC and D6 among endogenous chemokine receptors, see above) by internalizing the endogenous chemokine ligands that binds to the receptor and thereby removing the chemokines from the host cell surroundings as a way of evading the immune system, as discussed further below for the HCMV-encoded US28 [53, 124, 126, 127].

## 7. The C-Terminal Tail of Endogenous ****Chemokine Receptors

The endogenous chemokine receptors are rather similar in their C-terminal tails, and not different from the superfamily of endogenous class A 7TM receptors (Figure 3). The 19 human chemokine receptors have in average the same number of phosphorylation sites as the endogenous class A 7TM receptors (13 in each case), and an average length of 57 residues, which is in the proximity of the 66 residues in average for the endogenous class A receptors. The internalization routes and regulation has been described for several endogenous chemokine receptors, an interest facilitated by the discovery of CCR5 and CXCR4 acting as HIV cell-entry cofactors [125, 128, 129]. Consequently, the endocytosis pattern of CCR5 and CXCR4 and the regulation of this have been studied in great detail and it was recently shown that internalization of CXCR4 plays an important antiviral role [130–133]. In the case of CCR5, the binding of CCL5 leads to receptor phosphorylation of serine residues in the C-terminal tail by GRKs, which consequently leads to internalization and desensitization of the signal [134, 135]. Besides the involvement of the serine residues in *β*-arrestin recruitment, a dileucine motif in the C-terminal tail is also important for CCR5 receptor endocytosis [136]. Serial truncation of the CCR5 C-terminal tail resulted in progressive loss of cell surface expression, which could not be rescued by substitution with the C-terminal tail of CXCR4 [137]. Mutational analysis of CXCR4 showed that this receptor is likewise dependent on C-terminal serine phosphorylation sites and a dileucine motif for proper receptor internalization [138, 139]. Internalization of CXCR4 can follow two distinct pathways: CXCL12 ligand-mediated endocytosis was shown to be dependent on the serine phosphorylation sites whereas phorbol ester induced internalization is dependent on the dileucine motif [131, 132]. Interestingly, a naturally occurring mutation of CXCR4 with C-terminal deletions exists in patients with WHIM syndrome (warts, hypogammaglobulinemia, recurrent bacterial infection, myelokathexis). Loss of the C-terminal tail leads to decreased endocytosis of the receptor and consequently a reduced regulation of the receptor followed by an increased signaling with enhanced calcium flux and cell migration; a possible cause of the pathophysiology seen in WHIM syndrome [140]. Thus, the C-terminal tail plays an important role in the physiology of endogenous chemokine receptors.

## 8. The C-Terminal Tails of Virus-Encoded ****Receptors Are Generally Shorter

The HCMV-encoded chemokine receptor US28 signals constitutively via several pathways and upon stimulation by CC chemokines [54, 73]. Furthermore, CX3CL1 has been reported to act as an inverse agonist, albeit with low efficacy (up to 25% inhibition of basal activity) [141]. Additionally, unlike the majority of endogenous class A 7TM receptors, US28 is constitutively internalized in a ligand-independent manner [126, 127, 142]. Thus, by immunofluorescence staining, US28 was found to be accumulated intracellularly in endocytic organelles and by advanced immunogold electron microscopy shown to be localized to multivesicular endosomes [126]. Further studies revealed that US28 endocytosis occurs via a clathrin-mediated mechanism [127]. Importantly, only a small fraction of US28 is present at the cell surface (<20%), with the rest undergoing constitutive ligand-independent endocytosis with a fast internalization rate, as compared to CXCR4. Truncation of the C-terminal tail of US28 led to an increase in both magnitude and duration of the constitutive signaling indicating that the C-terminal tail plays a regulatory role in desensitizing the receptor. This was supported by hyperactivation of US28 in cells where *β*-arrestin 1 and 2 were genetically deleted [141]. A mutational analysis of serine residues in the C-terminal of US28 revealed that a decreased number of phosphorylation sites increased the cell surface expression of the receptor [143]. Truncating the C-terminal tail of US28 or replacing it with tails from other 7TM receptors (HHV8-ORF74 and human tachykinin NK1) led to an increase in constitutive activity of the receptor. Substitution of the HHV8-ORF74 tail with the tail from US28 diminished the cell surface expression of the HHV8-ORF74 chimera indicating that the C-terminal tail, in itself, is sufficient for desensitization by receptor endocytosis [144]. The constitutive endocytosis of US28 may serve as a chemokine scavenger and mediate the viral immune evasion by antagonizing the recruitment of cells involved in the immune response and thereby manipulating the host immune system. Another HCMV-encoded chemokine receptor, US27, also shows a large degree of intracellular localization. Swapping the C-terminal tail of US27 with that of the endogenous chemokine receptor CXCR3 led to cell surface expression similar to wild-type CXCR3; likewise, when substituting the endogenous tail with the viral US27 tail, the chimeric receptor was predominantly located intracellularly indicating that the C-terminal tail of US27 is necessary and sufficient for intracellular localization [145].

In general, the viral chemokine receptors, ORF74, encoded by several herpesviruses have very short C-terminal tails when compared to the endogenous receptors (Figure 3). A study of several *γ*-herpesviruses identified an eight-amino-acid conserved region at the membrane proximal part of the C-terminal tail suggested to play a role in G protein coupling and G_*α*_-selectivity [146]. Especially one basic residue showed importance for G_*αq*_ coupling—a residue which is conserved among the endogenous chemokine receptors suggesting an evolutionary conserved function of this residue such as G protein signaling [146]. HHV8-ORF74 is primarily located at the cell surface and deletion of the five terminal amino acids containing 3 phosphorylation sites did not seem to affect cell surface expression, though it did impact the signaling capabilities of the receptor seen by a diminished NF*κ*B and AP-1 signaling [147]. As signaling deficiencies are seen by the removal of just five amino acids, it is tempting to consider that the length of the C-terminal tail has been optimized to only contain necessities and thus demonstrating a minimum requirement for a functional viral receptor tail. Another study also found expression levels of 12 and 24 amino acids C-terminal tail deletions similar to wild-type albeit with reduced constitutive activity in spite of retained ligand regulation by chemokines [148]. It was suggested that the C-terminal helix 8, which is present in the terminal 24 amino acids, is involved in stabilizing the interaction between the receptor and G protein, thus playing a role in mediating signals upon chemokine binding [148]. Though the C-terminal tail of ORF74 appears to be involved in signal mediation, deletion of small parts of the tail including phosphorylation sites does not seem to largely affect cell surface expression or constitutive signaling suggesting that a short tail is enough for the receptor to function to a certain degree. Having such short tails might be a way of evading host regulatory mechanisms, such as GRKs and internalization, thereby ensuring virus-mediated constitutive signaling.

**Figure 4:**
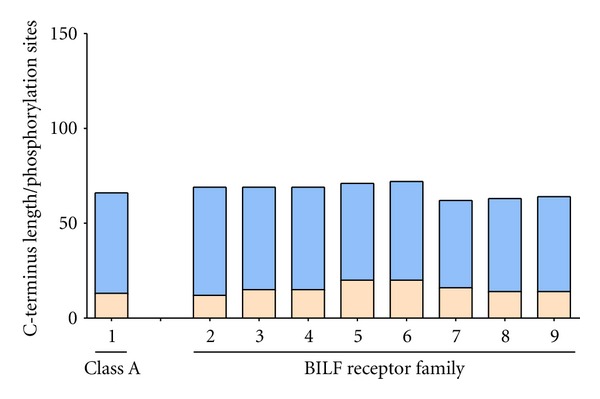
Relative sequence length and putative phosphorylation sites of the C-terminal region of class A 7TM receptors and the BILF-like receptors. The vertical axis displays the number of amino acids of the C-terminus (blue), defined as being after the highly conserved proline of the NPxxY-motif (class A 7TM receptors) or predicted by the Transmembrane Hidden Markov model using Geneious Pro (BILF receptor family), and the number of serine, threonine, and tyrosine in this region (beige). The horizontal axis displays the average of 334 non-olfactory class A 7TM receptors (1) and the EBV-encoded BILF receptors (2–9). For further details about specific receptors and GenBank accession number please see Table 2.

The BILF receptor family is rather conserved when it comes to the length of their C-terminal tails suggesting that the C-terminus serves an important purpose for the virus (Figure 4). The BILF1 receptor encoded by EBV shows a similar cell surface expression pattern as HHV8-ORF74 and signal constitutively through G_*α*i_ [80]. Though the BILF1 receptor does not resemble the endogenous chemokine receptors, it does serve a purpose in viral immune evasion as it is involved in internalization and degradation of MHC-I (major histocompatibility complex class I) molecules. Deletion of the C-terminal tail of the receptor led to impaired lysosomal degradation of internalized MHC-I molecules suggesting that the tail might contain a localization sequence guiding the receptor/MHC-I complex to the lysosomes [149].

## 9. Summary

From what is reviewed above, it is evident that the virus-encoded 7TM receptors differ from the endogenous counterparts—both from a structural and a functional point of view. The viral receptors have been captured from the host and through evolution (i.e., combinatorial chemistry by random mutagenesis followed by natural selection of the most virulent strain) been optimized to benefit the virus life cycle. As the chemokine receptor exploitation (and the general 7TM receptor piracy) is a widespread phenomenon among many viruses, it is likely that these receptors serve important purposes for virus survival, for instance, evasion of the antimicrobial immune response, viral persistence, viral dissemination, and control of own infection as shown for a few receptors. The selection of the chemokine system for interference by the viruses points towards this system as essential in multiple different immune responses. By studying the structural and functional alterations in the virus-encoded receptors as compared to the endogenous receptors, greater knowledge can be obtained for 7TM receptors in general. Thus, from a molecular pharmacology point of view, the chemokine receptors represent unique opportunities to study basic principles of receptor activation, internalization, and recycling pathways as examples of targeted evolution where the receptors have undergone major changes driven by a heavy evolutionary pressure. Since 7TM receptors are excellent drug targets, the development of high-potency antagonists or inverse agonists for the virus-encoded 7TM receptors could putatively pave the path for tomorrow's antiviral and anti-inflammatory drugs.

## Figures and Tables

**Table 1:** 

No.	Receptor	Accession number
1	Class A 7TM receptors	
2	CCR1	NP_001286.1
3	CCR2	NP_001116513.2
4	CCR3	NP_001828.1
5	CCR4	NP_005499.1
6	CCR5	NP_000570.1
7	CCR6	NP_004358.2
8	CCR7	NP_001829.1
9	CCR8	NP_005192.1
10	CCR9	NP_112477.1
11	CCR10	NP_057686.2
12	CCR11	NP_057641.1
13	CXCR1	NP_000625.1
14	CXCR2	NP_001548.1
15	CXCR3	NP_001495.1
16	CXCR4	NP_003458.1
17	CXCR5	NP_001707.1
18	CXCR6	NP_006555.1
19	CX3CR1	NP_001328.1
20	XCR1	NP_005274.1
21	HCMVUS28	P69332.1
22	RhCMVUS28.1	YP_068305.1
23	RhCMVUS28.2	AAN15199.1
24	RhCMVUS28.3	YP_068303.1
25	RhCMVUS28.4	YP_068302.1
26	RhCMVUS28.5	YP_068307.1
27	HCMVUS27	P09703.1
28	HCMVUL33	CAA37385.1
29	GpCMVGp33	AAK43591.1
30	RhCMVUL33	YP_068150.1
31	TCMVT33	NP_116383.1
32	MCMVM33	Q83207.1
33	RCMVR33	NP_064138.1
34	HHV6U12	P52380.1
35	HHV7U12	P52381.1
36	HHV6U51	NP_042944.1
37	HHV7U51	YP_073791.1
38	HHV8ORF74	AAC28486.1
39	MgHV68ORF74	NP_044914.1
40	AtHV3ORF74	NP_048046.1
41	SHV2ORF74	NP_040276.1
42	MMRVORF74	NP_570822.1
43	EHV2ORF74	NP_042670.1
44	EHV3E1	NP_042597.1
45	SPV 146 Swinepox virus	NP_570306.1
46	Sheeppox-GPCR	NP_659585.1
47	lumpy skin disease virus	AAN02735.1
48	Sheeppox-Q2/3L	Q86917.1
49	7L Yaba-like disease virus	NP_073392.1
50	7L Yaba Monkey Tumor virus	NP_938268.1
51	145R-Yaba Monkey Tumor virus	NP_938396.1
52	145R-Yaba-like disease virus	NP_073530.1

**Table 2:** 

No.	Receptor	Accession number
1	Class A 7TM receptors	
2	CaHV3-ORF6	NP_733858.1
3	CHV15-BILF1-rh	YP_068006.1
4	EBV-BILF1	YP_401711.1
5	EHV2-E6	NP_042607.1
6	AlceHV-E5	NP_065513.1
7	PLHV3-A5	AAO12316.1
8	PHV2-A5	AAF16523.1
9	PLHV1-A5	AAF16521.1
